# Interest in Fertility Preservation Among Adults Seen at a Gender Care Clinic

**DOI:** 10.3390/jcm14176175

**Published:** 2025-09-01

**Authors:** Quinnette Jones, Scott M. Carlson, Shilpi Agrawala, Andrew Weinhold, Heather E. Parnell, Katelyn M. Holliday, Carly E. Kelley

**Affiliations:** 1 Department of Family Medicine & Community Health, Division of PA Studies, Duke University, 800 S. Duke Street, Durham, NC 27701, USA; quinnette.jones@duke.edu; 2 Department of Medicine, Division of Endocrinology, Metabolism, and Nutrition, Duke University, 30 Medicine Circle, Durham, NC 27710, USA; 3 Department of Endocrinology, Baylor University Medical Center, Dallas, TX 75246, USA; 4 Department of OBGYN, Division of Reproductive Endocrinology and Infertility, Duke Fertility Center, 5601 Arringdon Park Drive, Suite 210, Morrisville, NC 27560, USA; 5 Dallas IVF, Dallas, TX 75246, USA; 6 Duke Global Health Institute, Center for Health Policy and Inequalities Research, Duke University, 310 Trent Dr, Durham, NC 27710, USA; 7 Department of Family Medicine and Community Health, Duke University, 2200 W. Main Street, Suite 400, Durham, NC 27705, USA

**Keywords:** transgender, gender-affirming care, fertility preservation

## Abstract

**Introduction/Background**: Medical treatments received by transgender and/or gender diverse (TGD) people can impact fertility, yet the literature lacks data on factors that influence fertility decisions among TGD people. **Specific Aim(s):** This study aimed to identify predictors of interest in fertility preservation (IFP). **Materials and Methods:** This retrospective observational study utilized data from 2021–2023 from an adult gender registry for patients receiving care at academic medical center (n = 206). Patient demographic data and survey responses to questions about fertility were queried and analyzed. Bivariate and multivariate analyses were conducted using logistic regression. **Results:** Most patients (73.8%, n = 152) were not interested in fertility preservation (FP) and 16.5% (n = 34) were unsure. Reasons most often cited were not wanting biological children (55.9%, n = 104), preferring adoption (20.4%, n = 38), cost (19.9%, n = 37), and dysphoria (19.4%, n = 36). Bivariate analyses showed that increasing age, being married, and already having children were significantly inversely associated with IFP (*p* = 0.03, 0.01, 0.02, respectively). Non-Hispanic Black race/ethnicity (OR (95% CI): 3.43 (1.19, 9.84)) and disability or unemployment (OR (95% CI): 4.19 (1.42, 13.00)) were significantly associated with IFP vs. Non-Hispanic White race/ethnicity and full-time employment, respectively. In multivariate models, being married was significantly inversely associated with IFP, e.g., OR (95% CI): 0.30, (0.07, 0.99), when accounting for age and already having children. Race/ethnicity and employment comparisons remained significant after adjusting for other factors. **Conclusions:** Most patients did not desire FP. Among those IFP, potential predictors include age, marital status, already having children, race and ethnicity, and employment and disability status.

## 1. Background

There are 1.6 million transgender and gender diverse (TGD) people in the United States (US), and that number is growing as the proportion of individuals who identify as TGD is increasing among younger generations [1]. The demand for gender-affirming medical treatment is thus increasing, with a trend toward decreasing age at time of presentation [2]. Gender-affirming treatments, including gender-affirming hormone therapy (GAHT) and gender-affirming surgery (GAS), significantly impact fertility potential and outcomes. The extent to which the effects of GAHT on fertility are reversible is not fully understood, while GAS with gonadectomy results in permanent sterility.

Several previous studies have shown a high reproductive desire among TGD individuals, yet the rate of fertility preservation (FP) remains low, despite a relative increase in access to reproductive counseling and resources [3,4,5]. The Endocrine Society, American Society for Reproductive Medicine, and World Professional Association for Transgender Health all recommend reproductive and fertility counseling for TGD patients before initiating GAHT [6,7,8]. Given the potential impacts of GAHT and gonadectomy on FP, along with the potential need to temporarily stop GAHT to preserve fertility, providing education and managing treatment expectations is essential. Furthermore, TGD individuals should be provided with appropriate referrals for family planning and FP if desired. Though little has been published regarding optimizing this counseling and referral process, some studies examining FP rates in TGD individuals have attempted to elucidate why most choose not to preserve fertility prior to GAHT. These studies have primarily been conducted in non-US [9,10,11,12] pediatric and adolescent [13,14,15], and/or exclusively transgender male populations [13,16].

A study of TGD adults in the US described fertility desires and perceived barriers to FP among patients seeking care at a multidisciplinary university-based gender clinic in California. Patients who were undecided about FP prior to transition expressed moderate to severe decisional regret, and 37% of the total study population stated that their family planning goals were not adequately addressed at their initial visit [17]. Only two percent of patients requested referral for FP at the initial visit, with reported barriers to FP being cost, need to delay/discontinue GAHT, and concerns for worsening gender dysphoria [17]. Interestingly, this percentage increased to 33% at subsequent follow-up, a finding that is reflective of evolving reproductive priorities with age and comparable to the 37.5 to 51% of TGD adults in European studies who would have considered FP before GAHT if offered [17,18]. Another US multi-site cross-sectional study in Chicago found that fertility desires correlated with an increased willingness to pause GAHT, and cost was the greatest barrier to pursuing FP [19].

A lack of desire for biological children is another reason TGD patients may not pursue FP. A recent systematic review of studies from 16 different countries showed that while there is a high desire for children among TGD adults, the desire for biological children was lower [20]. The largest US study of TGD patient reproductive desires to date, a single-centercohort (n = 255), showed even lower rates of desiring biological children among TGD individuals and higher rates of uncertainty [21]. The desire for a biological child in this cohort was similar among the various age groups between 16 and 39 years, while almost half of the individuals over 40 years already had a biological child, and none of the individuals over 40 years desired a child or additional child. The overall rate of FP in this study was low at 2% for individuals designated female at birth (DFAB) and 8% for individuals designated male at birth (DMAB) [21].

Building upon this prior research, the present study aims to further characterize FP interest among TGD individuals and to explore potential barriers to pursuing FP. Specifically, we sought to compare demographic and clinical characteristics between patients who expressed interest or uncertainty regarding FP (IFP) and those who were not interested (NFP), with the goal of identifying factors that may predict a lack of desire for FP. These findings may inform future strategies for counseling and referral processes in gender-affirming care.

## 2. Materials and Methods

### 2.1. Study Design

We conducted a retrospective observational study of adult TGD patients enrolled in the Duke Research for Equitable Access to Medicine (DREAM) Registry who completed questions regarding FP on standard gender care clinic intake forms between March 2021 and October 2023.

### 2.2. Study Sample

Participants were selected using consecutive inclusion from the DREAM Registry; all individuals meeting eligibility criteria and presenting to the gender care clinic between March 2021 and October 2023 were enrolled. Eligibility criteria included age ≥ 18 years, completion of an initial clinic visit within the study period, completion of the clinic intake form including FP questions, and provision of informed consent to participate in the DREAM Registry.

### 2.3. Data Sources

The DREAM Registry started in March 2021 and comprises TGD adults receiving care within the Duke Gender Medicine Clinic since its January 2018 inception. DREAM includes both patient-reported and electronic medical record data and seeks to better understand patient needs, preferences, and short and long-term health outcomes of GAHT. Patients receive surveys to complete prior to their visits that collect information on the following: demographics, sexual orientation and gender identity, medical, surgical, mental health, preventive care, substance use, sexual, and reproductive histories and goals, FP, transition goals, and past preventive care screening (see Supplementary Materials). New patients are invited to participate in the registry upon intake, while established patients were invited to the registry during their first return visit after the registry launched. Informed consent is obtained, and data are collected at baseline, 6 months, 12 months, 18 months, 24 months, and then annually. DREAM was approved by the Duke Health Institutional Review Board (protocol numbers: Pro00105329 and Pro00112889).

### 2.4. Measures/Variables

The primary outcome was participant interest in FP. This was measured by participant responses to survey questions that asked participants to respond “yes”, “unsure”, or “no” to whether they are interested in FP. Those who responded “unsure” or “no” were asked further questions about why they are unsure or NFP. For the purposes of analysis, “yes” and “unsure” responses were grouped as IFP, and “no” responses were grouped as NFP.

Demographic variables were self-reported and included age in years (18–21, 22–26, 27–34, and ≥35), designated sex at birth (DSAB) (female, male), self-identified gender identity (transgender male+, transgender female+, non-binary/genderqueer), self-identified sexual orientation (asexual, bisexual, gay, lesbian, do not label sexuality, pansexual, queer, straight/heterosexual, not listed, more than one selected, choose not to disclose), race and ethnicity (Hispanic Latino, Non-Hispanic Asian, Non-Hispanic Black, Non-Hispanic Other, Non-Hispanic White, missing), education (≤high school or GED, some college or Associates, Bachelors, Master’s or PhD, missing), and employment status (full-time, part-time, student, retired, disability or unemployed, missing).

Additional participant characteristics included in the analyses were: GAHT at baseline (yes, no, missing), a history of suicidal plans or attempts (yes, no, missing), PROMIS Emotional Support score (<50, ≥50, missing), parental support of gender identity (yes, no, missing), marital status (married, not married, missing) and whether or not the participant already has children (yes, no). The PROMIS Emotional Support instrument measures feelings of being cared for and valued. The general US population mean on the PROMIS is 50, with higher scores indicating better than average emotional support [22].

### 2.5. Analyses

Associations between potential predictor variables and desired FP (IFP vs. NFP) were examined using Pearson Chi-sq and Fisher exact tests for binomial and nominal predictors and Cochran–Mantel–Haenszel for ordinal predictors (age category), as well as with bivariate logistic regression. Potential predictors with *p*-values < 0.1 were carried forward for multivariate logistic regression modelling. Due to the limited sample size, models could only accommodate up to four simultaneous predictors. Exact logistic regression was used for variables with sparse cells (<5 counts). Analyses were considered exploratory and did not adjust for multiple comparisons. Analyses were implemented using SAS software, Version 9.4 (SAS Institute Inc., Cary, NC, USA).

## 3. Results

### 3.1. Sample Demographics

**Figure 1:**
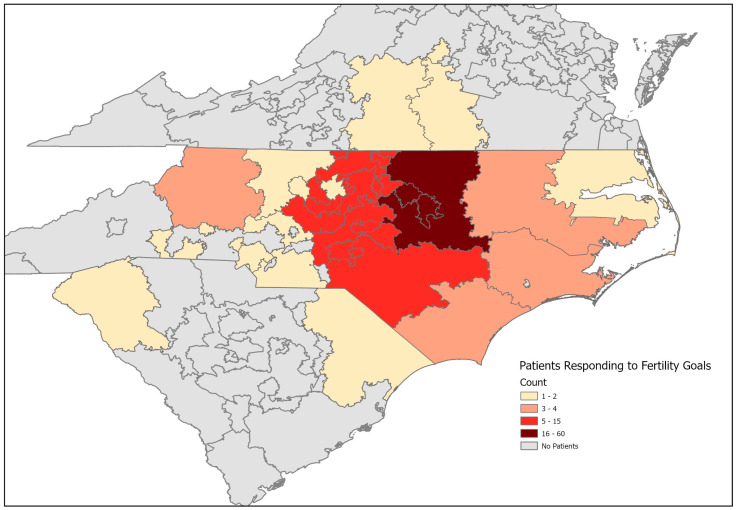
Count of included DREAM Registry patients residing in geographic areas defined by the first 3 digits of the zip code. n = 1 patient from a non-contiguous state not shown.

Of 240 eligible participants, 217 (90%) completed intake questions related to reproductive history and fertility plans. Individuals with actual or perceived pre-existing infertility were excluded based on the following criteria, leaving 206 participants for analysis: (1) age > 55 for those assigned female at birth (n = 2) and (2) sole provided rationale for not wanting to preserve fertility indicated perceived pre-existing infertility (n= 9; e.g., “sterile”, “infertile”, “puberty blocker used”). Although patients came from a single clinic, the clinic has a broad reach across the state, including patients from outside of the state (Figure 1).

The median (interquartile range) age of respondents was 27 (22–35) years (overall range: 18–78 years), with 54.9% of participants DFAB and 45.1% DMAB. Participants selected 0 to 4 gender identities, with 74.1% selecting a single identity. When grouped as “transgender male”, “transgender female”, and “non-binary or genderqueer or genderfluid”, the most prevalent gender identities were transgender female (41%), transgender male (39%), and gender non-binary or genderqueer (20%). For sexual orientation, participants selected 0 to 5 sexual orientations, with 70.9% of those who selected an orientation selecting only one, and 24.5% selecting more than one sexual orientation. Singly selected sexual orientations with the highest prevalence were queer (13.6%), pansexual (13.6%), do not label sexuality (12.5%), straight/heterosexual (9.8%), and bisexual (8.2%). When individuals selected more than one sexual orientation, they most often selected queer along with one or more other sexual orientations (73.3%). In general, TGD patients appeared to view their sexual orientation in relation to their gender identity and not sex at birth, i.e., 61.5% of patients who selected lesbian were transgender female (vs. 15.4% transgender male and 23.9% non-binary) and 64.7% who selected gay were transgender male (vs. 29.4% transgender female and 5.9% non-binary). However, 68.4% of those who selected straight/heterosexual were transgender male vs. only 15.8% transgender female (and 15.8% non-binary or genderqueer/fluid). For race and ethnicity, most participants were Non-Hispanic White (76.8%), followed by Non-Hispanic Black (10.8%) and Hispanic Latino (7.2%). The most frequently reported education levels were some college or associate degrees (41.3%), bachelor’s degrees (24.0%), and high school or less (21.2%). When asked about employment, 37.2% reported full-time and 16.4% reported part-time employment, 25.1% reported they were students, 18.0% reported disability or unemployment, and 3.3% were retired. At the time of baseline survey completion, 35.1% of participants reported they were already taking GAHT

### 3.2. Psychological and Social Support Measures

There are several psychological and social factors to consider when investigating FP preferences. Mean PROMIS Emotional Support scores were higher than or equal to the general population mean of ≥50 for 67.1% of participants [22]. Most participants (54.2%) reported that their parents are not supportive of their gender identity. When asked if they had ever planned or attempted to kill themselves, 36.4% of participants reported “yes”. For marital status, 78.7% of participants reported they were not married. Most participants (81.6%) reported that they did not already have biological children.

### 3.3. Participant Characteristics and Fertility Preferences

Most participants (73.8%, n = 152) reported “no” (NFP) when asked if they were interested in preserving their fertility, 9.7% (n = 20) said “yes”, and 16.5% (n = 34) said “unsure” to this question. In contingency table analyses, there was no association between DSAB, gender identity, current GAHT use, previous suicide plan or attempt, or parental support of gender identity and fertility desires (Table 1). For example, 53.7% of those IFP were DFAB vs. 46.3% DMAB (*p* = 0.8). However, there were significant or suggestively significant associations between age category, sexual orientation, race and ethnicity, education, employment, PROMIS score category, marital status, and having already had children and fertility desires. For example, the association between employment status and fertility preference was statistically significant (*p* = 0.02), with those responding IFP largely comprised of students (31.1%, n = 14) and those on disability or unemployed (28.9%, n = 13). Statistically significant associations between marital status (*p* = 0.003) and reporting already having children (*p* = 0.01) were also identified. Those who responded with IFP were more often unmarried (92.5%, n = 49) than married (7.6%, n = 4) and more often had no current children (92.6%, n = 50) than already had children (7.4%, n = 4).

### 3.4. Reasons for Electing Not to Pursue FP

Participants who indicated that they were unsure about or did not want to pursue FP were able to select one or more of the following reasons they would not pursue FP: “too expensive”, “do not want biological children”, “prefer to adopt”, “hope to still have biological children even after hormones are started”, “FP will cause too much distress and worsen dysphoria”, or “not listed”. “Not wanting biological children” was the most selected reason (55.5%; n = 104) by those indicating no or unsure of interest in FP followed by prefer to adopt (20.4%, n = 38), too expensive (19.9%, n = 37), and distress/worsen dysphoria (19.4%, n = 36). Participants were able to write in reasons not listed for not wanting or being unsure about FP. Of the 18.8% (n = 35) written in answers, already having children (9.1% of total, n = 17) was the most frequent response followed by old age (2.7% of total, n = 5) and their partner would carry/provide eggs or they themselves had already preserved fertility (2.2% of total, n = 4). See Table 2.

### 3.5. Predictors of IFP

Table 3 presents the results of bivariate logistic regression analyses. Results showed that increasing age, being married, and already having children were inversely associated with desiring FP. The odds of desiring FP decreased 0.83 per five-year increase in age (OR (95% CI): 0.83 (0.70, 0.98)). Marital status was the strongest overall predictor, with the odds of desiring FP among those who were married as 0.23 times the odds of those who were not married (OR (95% CI): 0.23 (0.06, 0.69)). The odds of desiring FP among those who already had children were 0.28 times the odds of those who did not have children (OR (95% CI): 0.28 (0.07, 0.84)). Race and ethnicity and employment status were not significantly associated as overall variables; however, specific comparisons were statistically significant. The odds of desiring FP among Non-Hispanic Black patients were 3.43 times the odds among Non-Hispanic White patients (OR (95% CI): 3.43 (1.19, 9.84)). The odds of desiring FP among those who were disabled or unemployed were 4.19 times the odds among those who were employed full-time (OR (95% CI): 4.19 (1.42, 13.00)) and the odds of desiring FP among students were 2.84 times the odds among those who were employed full-time (OR (95% CI): 2.84 (1.02, 8.34)). Logistic regression could not be completed for sexual orientation due to the large number of categories with small cell sizes. Table 4 presents the results of the multivariate FP desires models built from potential predictors identified in Table 1 and Table 3. The models were only able to accommodate up to four variables at a time due to sample size restrictions. In Models 1–4, marital status was most consistently associated with IFP. For example, the odds of desiring FP among those who were married were 0.3 times the odds among those who were not married (OR (95% CI): 0.30 (0.07, 0.99)) after accounting for age and already having children. In full models (Models 5–8), the previously noted specific significant comparisons for race and ethnicity and employment remained after adjusting for age, marital status, and already having children: (1) Non-Hispanic Black race and ethnicity was significantly associated with IFP as compared with Non-Hispanic White (OR (95% CI): 3.06 (1.04, 9.02)) and (2) reporting disability or unemployment was significantly associated with IFP vs. full-time employment (OR (95% CI): 4.10 (1.37, 12.88)).

### 3.6. Referrals to Fertility Preservation and Fertility Preservation Rates

Among 54 individuals IFP, 15 (28%) were referred to urology or reproductive endocrinology and infertility (REI). Eight (15%) ultimately attended an appointment, and only six (11%) pursued FP. One individual pursued cryopreservation through a third-party online fertility service. The overall rate of FP among the entire cohort was 3% (n = 6/206).

## 4. Discussion

Data on the characteristics of TGD adults and their interest in FP in the US are limited. This study contributes to the growing body of literature by offering new insights into the associations between individual characteristics and interest in FP, as well as identifying potential reasons for not pursuing FP among TGD patients. Notably, over 26% of participants in our cohort expressed potential interest in FP, a proportion higher than that reported in previous US-based studies [21]. As the number of TGD individuals in younger age groups continues to rise, greater interest in FP may be expected. Our study found that IFP was comparable between people DFAB and DMAB, whereas prior studies have reported lower interest among people DFAB [20,21]. The overall rate of FP, however, was low at 3%, consistent with prior studies [17,20]. This low percentage may not fully capture the range of reproductive intentions, as some individuals may choose not to undergo formal FP but still pursue biological parenthood by discontinuing GAHT when they are ready to conceive. Nevertheless, the discrepancy between interest in FP and the low rate of completed procedures suggests that barriers remain in accessing or pursuing fertility services.

Interestingly, being unmarried was consistently associated with an increased odds of IFP across all models. One possible explanation for the association between unmarried status and greater interest in FP among TGD individuals is that unmarried individuals may be more likely to consider future reproductive possibilities as part of family-building and long-term life goals, especially if they anticipate future partnership or parenthood. In contrast, married individuals may have already made reproductive decisions with their partners, including the decision to have children, and may feel a greater sense of resolution regarding future childbearing, having aligned their goals within the context of the relationship. The decreased odds of IFP among participants with biological children in our cohort further support this interpretation. Prior negative parenting experiences may also partially explain the reduced interest in FP among TGD adults who already have biological children. Studies have shown that the majority of TGD parents started parenting before their gender transition, and many of them experienced discrimination and reduced parental rights because of their gender identity [20]. Fear of stigmatization, discrimination, and adverse reactions regarding the desire for biological children have been shown to negatively impact sexual and gender minority peoples’ family planning decisions [23] and are likely contributors to overall interest in FP. However, no patients in the DREAM Registry listed this as a reason for not desiring FP.

Our study showed that increasing age was a predictor of decreasing IFP. People over the age of 35 had the least IFP, a finding that is consistent with prior research [21]. We additionally found increased odds of IFP among people who were Non-Hispanic Black. The intersectionality of being TGD and a racial/ethnic minority has been shown to compound barriers to accessing healthcare, including gender-affirming and reproductive services. This highlights the critical need for targeted support and tailored interventions to help TGD patients successfully navigate the healthcare system when FP is desired [18].

Moreover, our findings revealed increased odds of IFP among participants who reported being unemployed or on disability. This association between IFP and employment status may reflect, in part, a greater availability of time and flexibility to focus on future family planning and parenting considerations. It may also indicate differing priorities or social support within this group. For example, more frequent interactions with healthcare or social support services could increase awareness of fertility options and access to counseling. Some individuals may have delayed FP due to prior health or social challenges and are now expressing greater interest as their circumstances stabilize; they may alternatively view parenthood as a source of hope and stabilization for the future. Further research is needed to explore the underlying factors driving this association and to better understand how socioeconomic status intersects with reproductive goals among TGD individuals.

Reasons identified in our study for NFP are consistent with findings from prior research and include not desiring biological children, a preference to adopt, cost, the potential for worsening dysphoria, and already having biological children. Some participants hoped for children even after starting GAHT, while some reported that they had already done FP or that their partner would assume childbearing responsibilities. NFP in our study may also be partially attributable to a larger societal trend in the general population. Interest in having children appears to be declining notably, particularly among younger adults under age 50 [24]. Voluntary childlessness is increasing, with nearly one-third of nonparents in 2023 expressing no desire ever to have children, more than double the rate from 2002 [25]. Among those who indicated that they were not planning to have children, the majority (57%) simply did not want to have children [26].

Given that the barriers to FP or the desire to have biological children may change over time, it is important to ensure that counseling is tailored to patient goals and explored routinely within the context of a gender care visit. Furthermore, FP counseling should consider that the feasibility and burden of FP differ significantly based on whether a person is freezing sperm or oocytes. For those DMAB, sperm cryopreservation requires discontinuation of estradiol, and the return of viable sperm in semen analysis may take several months, contributing to significant dysphoria. If well-coordinated referral systems are in place, this process can be completed relatively quickly, within 24 to 72 h, prior to initiation of GAHT. For those DFAB, oocyte cryopreservation typically requires a two-week cycle of ovarian stimulation, and though testosterone is often paused, there are case reports of successful egg retrieval for patients remaining on testosterone, depending on ovarian reserve and age [23,24]. The financial burden differs substantially, with sperm cryopreservation for those DMAB generally costing $750–$1000 in the US, whereas egg freezing for those DFAB can exceed $10,000 per cycle, not including annual storage fees, posing a greater barrier to equitable access. Overall, these medical, emotional, and financial factors underscore the need for proactive, patient-centered fertility referrals and counseling.

Patients undergoing gonadotoxic treatments for cancer treatment are similarly faced with decisions about FP and oncology care guidelines (ASCO and ASRM) include FP counseling and referrals for reproductive-aged patients [27]. Despite this, FP remains infrequently utilized among US cancer patients, even when discussing eligibility and availability. Overall, utilization is higher among younger patients and those with private insurance, but remains below 15% in most groups [27,28,29]. A US study of female patients found that only 6–7% of those aged 18–35 underwent FP procedures, with the rate dropping sharply to just 0.3% among individuals aged 41–45 [27]. Another study of female cancer patients found that older age at diagnosis, non-Hispanic Black race, having children, residing in non-urban areas, and lower socioeconomic status were all associated with a decreased likelihood of pursuing FP [28]. Rates of sperm cryopreservation among males with cancer are slightly higher at 9% [29]. However, among those who bank sperm, actual use of stored material is low, with long-term usage rates under 15% [29]. These findings highlight persistent gaps between counseling, FP, and follow-through, paralleling challenges seen in both oncology and TGD patient populations, and underscore the need for improved access, financial support, and provider education across disciplines.

Oncology has made more structured advances in integrating FP into patient care, and in this respect, is ahead of current TGD care pathways. In oncology, clinical guidelines mandate referrals for FP, ensuring more consistent access and awareness, whereas TGD care guidelines typically only recommend discussion of FP, which may result in less systematic follow-through. Additionally, many oncology programs benefit from dedicated onco-fertility services and multidisciplinary teams that bridge oncology and reproductive care, a model that remains rare within the TGD care setting. Insurance coverage is another important variable, as many states have mandates requiring insurers to cover FP when infertility is caused by medical treatments like chemotherapy, aligning with ASCO and ASRM guidelines [30]. In contrast, there are no state mandates requiring coverage of FP for TGD undergoing gender-affirming care, and most insurance plans exclude it. As a result, TGD patients often face the full out-of-pocket cost, while oncology patients are more likely to receive coverage, especially in states with FP laws.

Overall, the strengths of our study were its relatively large sample size and the ability to assess patient characteristics, desire for FP, and reasons for not wanting to pursue FP. A primary limitation is the single-center study design within a specialized academic gender care clinic that included a primarily White and educated cohort, which is not representative of the larger, more diverse US population. Nevertheless, this clinic has a broad geographic reach across the state and increases the geographic diversity represented in the literature. A second limitation is that we were not able to account for potential structural and interpersonal barriers, including lack of insurance coverage, overall cost, and public funding differences between sperm and egg cryopreservation, and procedural logistics [31]. A third limitation is the lack of detailed information and documentation beyond what was reported with the initial survey questions. For example, we did not have the ability to ask patients whether they felt adequately educated or counseled about FP during their gender care visits. Our study also did not evaluate patients’ feelings toward fertility at subsequent visits, which may change after initiation of GAHT. Finally, given our study design, we could not compare interest in FP or reasons for NFP between TGD individuals and the general population.

## 5. Conclusions

Our study showed that about one quarter of TGD adults seeking GAHT at a US academic gender care clinic were IFP, but the overall rate of pursuing FP is much lower. Unmarried status, absence of biological children, unemployment or disability, and Black Non-Hispanic race/ethnicity increased the odds of being IFP, while increasing age decreased the odds of FP. These findings help provide additional insights into the fertility decisions of TGD patients and the potential barriers to FP. Information gained from this study, such as the increased interest among Black Non-Hispanic TGD adults, creates an opportunity to develop strategies to better support patients interested in FP.

Larger multi-site cohort studies are needed to improve our understanding of the specific concerns and needs of TGD patients who may or may not wish to pursue FP. Qualitative, interview-based studies of TGD experiences are needed to guide interventions targeting the standardization of fertility counseling and education. Research should aim to reduce the structural and interpersonal barriers to FP and improve care coordination for those who wish to have biological children. Improving the scope of education materials and tailoring content to the special considerations of DSAB and gender identity groups may also aid in clarifying the desire for FP and/or completion of the process.

## Figures and Tables

**Table 1:** Participant Characteristics Overall and by Fertility Preferences.

		Sample		Interested in Fertility Preservation				
		Total (n = 206)		Yes/Unsure (n = 54)		No (n = 152)		
		n	%	n	%	n	%	*p*-Value ^11^
Age	18–21	50	24.3	13	24.1	37	24.3	0.1
	22–26	53	25.7	19	35.2	34	22.4	
	27–34	51	24.8	14	25.9	37	24.3	
	≥35	52	25.2	8	14.8	44	29.0	
Designated Sex at Birth	Female	113	54.9	29	53.7	84	55.3	0.8
	Male	93	45.2	25	46.3	68	44.7	
Gender Identity ^1^	Transgender Male+ ^2^	80	38.8	22	40.7	58	38.2	0.9
	Transgender Female+ ^3^	84	40.8	21	38.9	63	41.5	
	Non-binary/Genderqueer ^4^	42	20.4	11	20.4	31	20.4	
Sexual Orientation ^5^	Asexual	8	4.4	1	2.1	7	5.1	0.06
	Bisexual	15	8.2	1	2.1	14	10.2	
	Gay	7	3.8	2	4.3	5	3.7	
	Lesbian	11	6.0	6	12.8	5	3.7	
	Do not label sexuality	23	12.5	10	21.3	13	9.5	
	Pansexual	25	13.6	7	14.9	18	13.1	
	Queer	25	13.6	6	12.8	19	13.9	
	Straight/Heterosexual	18	9.8	2	4.3	16	11.7	
	Not listed	7	3.8	3	6.4	4	2.9	
	More than one selected	45	24.5	9	19.2	36	26.3	
	Choose not to disclose/Missing ^6^	22	-	5	-	17	-	
Race and Ethnicity	Hispanic Latino	14	7.2	4	8.3	10	6.9	0.08
	Non-Hispanic Asian	4	2.1	1	2.1	3	2.1	
	Non-Hispanic Black	21	10.8	10	20.8	11	7.5	
	Non-Hispanic Other ^7^	6	3.1	2	4.2	4	2.7	
	Non-Hispanic White	149	76.8	31	64.6	118	80.8	
	Missing	12	-	6	-	6	-	
Education	≤High School or GED	38	21.2	7	15.2	31	23.3	0.06
	Some College or Associates	74	41.3	27	58.7	47	35.3	
	Bachelors	43	24.0	8	17.4	35	26.3	
	Master’s or PhD	24	13.4	4	8.7	20	15.0	
	Missing	27	-	8	-	19	-	
Employment	Full-Time	68	37.2	9	20.0	59	42.8	0.02
	Part-Time ^8^	30	16.4	9	20.0	21	15.2	
	Student ^9^	46	25.1	14	31.1	32	23.2	
	Retired	6	3.3	0	0.0	6	4.4	
	Disability or Unemployed	33	18.0	13	28.9	20	14.5	
	Missing	23	-	9	-	14	-	
HRT	Yes	72	35.1	16	29.6	56	37.1	0.3
	No	133	64.9	38	70.4	95	62.9	
	Missing	1	-	0	-	1	-	
Suicide Plan or Attempt	Yes	72	36.4	22	41.5	50	34.5	0.4
	No	126	63.6	31	58.5	95	65.5	
	Missing	8	-	1	-	7	-	
PROMIS category	<50	51	32.9	19	44.2	32	28.6	0.1
	≥50	104	67.1	24	55.8	80	71.4	
	Missing	51	-	11	-	40	-	
Parents Support Identity	Yes	94	45.9	22	40.7	72	47.7	0.4
	No	111	54.2	32	59.3	79	52.3	
	Missing	1	-	0	-	1	-	
Marital Status	Married	43	21.3	4	7.6	39	26.2	0.003
	Not Married	159	78.7	49	92.5	110	73.8	
	Missing	4	-	1	-	3	-	
Already has children ^10^	Yes	38	18.5	4	7.4	34	22.4	0.01
	No	168	81.6	50	92.6	118	77.6	

^1^ Participants could select all that apply: female (cis gender), genderqueer or genderfluid, male (cis gender), non-binary, transgender male, transgender female, choose not to disclose, not listed with write in option; ^2^ Transgender male+ defined as: Transgender male alone (n = 54) or in combination with another non-transgender female identity (n = 20); Male (cis gender) combined with DFAB (n = 1); write-in option indicating transgender male (n = 2); or missing/undisclosed and assigned by medical record (n = 3). ^3^ Transgender female+ defined as: Transgender female alone (n = 59) or in combination with another non-transgender male identity (n = 20); Female (cis gender) combined with DMAB (n = 4); or write-in option indicating transgender female (n = 1). ^4^ Non-binary, genderqueer, or genderfluid; alone (n = 27 and n = 5), in combination with each other (n = 6), or in combination with a cis gender matching sex at birth, a write-in option not indicating transgender, or both male and female genders (n = 4). ^5^ Participants could select more than one option, only n = 45 did. ^6^ Choose not to disclose (n = 7); Chose no options/missing (n = 15). ^7^ Non-Hispanic Other includes American Indian or Alaskan Native, Asian, Asian Indian, Chinese, Filipino, Guamanian or Chamorrro, Japanese, Korean, Native Hawaiian, Not Reported/declined, Other, Other Asian, Samoan, Vietnamese, (n = 3); or more than one race (n = 3). ^8^ Includes n = 9 who only selected “self-employed” and not “full-time” or “part-time”. ^9^ Regardless of being employed full-time, part-time, or unemployed. ^10^ Due to the way the question was asked (“If you have children, what are their ages?”), this may be an undercount of the number of individuals who already have children, particularly if they are adult children. We have added n = 5 to the “Yes” category based on their write-in response of already having children as their reason for not wanting to preserve fertility. All 5 were older individuals (58+) who did not list the age of the child. There is also no way to distinguish between “No” and “Missing”. ^11^ Pearson Chi-sq or Fisher exact for binomial and nominal predictors; CMH for ordinal predictors (age category).

**Table 2:** Possible Reasons for Not Wanting to Preserve Fertility among those Responding No or Unsure to Fertility Preservation (n = 186).

Reason	n	%
Too Expensive	37	19.9
Do not want bio kids	104	55.9
Prefer to adopt	38	20.4
Hope to still have despite HRT	9	4.8
Distress/Worsen Dysphoria	36	19.4
Not listed (write in)	35	18.8
Write in: Have kids already	17	9.1
Write in: Older age-related	5	2.7
Write in: Partner/Preserved *	4	2.2

* Participant indicated their spouse/partner would carry/provide eggs (n = 3) or that they had already preserved their own fertility (n = 1).

**Table 3:** Bivariate Associations between Fertility Preservation Desires ^1^ and Predictor Variables.

		OR ^2^	95% CI	*p*-Value
Age	Per 5-year increase	0.83	(0.70, 0.98)	0.03
Designated Sex at Birth	Female	1		
	Male	1.07	(0.57, 1.99)	0.8
Gender Identity	Non-binary/Genderqueer	1		
	Transgender Female+	0.94	(0.40, 2.19)	0.9
	Transgender Male+	1.07	(0.46, 2.49)	
Race and Ethnicity	Non-Hispanic White	1		
	Non-Hispanic Asian	1.27	(0.02, 16.41)	1.0
	Non-Hispanic Black	3.43	(1.19, 9.84)	0.02
	Non-Hispanic Other	1.89	(0.16, 13.93)	0.8
	Hispanic Latino	1.52	(0.33, 5.73)	0.7
Education	≤High School or GED	1		
	Some College or Associates	2.52	(0.92, 7.73)	0.08
	Bachelors	1.01	(0.28, 3.70)	1.0
	Master’s or PhD	0.89	(0.17, 4.05)	1.0
Employment	Full-Time	1		
	Part-Time	2.78	(0.85, 9.13)	0.1
	Student	2.84	(1.02, 8.34)	0.05
	Retired	0.85	(0.00, 4.92)	0.9
	Disability or Unemployed	4.19	(1.42, 13.00)	0.01
HRT	No	1		
	Yes	0.71	(0.37, 1.40)	0.3
Suicide Plan or Attempt	No	1		
	Yes	1.35	(0.71, 2.57)	0.4
PROMIS category	<50	1		
	≥50	0.51	(0.24, 1.05)	0.07
Parents Support Identity	No	1		
	Yes	0.75	(0.40, 1.42)	0.4
Marital Status	Not Married	1		
	Married	0.23	(0.06, 0.69)	0.005
Already has children	No	1		
	Yes	0.28	(0.07, 0.84)	0.02

^1^ Desire or unsure about fertility preservation vs. do not desire. ^2^ Exact logistic regression used when warranted due to small cell size.

**Table 4:** Multivariate Associations between Fertility Preservation Desires ^1^ and Predictor Variables.

		OR ^2^	95% CI	*p*-Value
MODEL 1				
Age	Per 5-year increase	0.91	(0.76, 1.08)	0.3
Marital status	No	1		
	Yes	0.29	(0.07, 0.98)	0.05 *
MODEL 2				
Age	Per 5-year increase	0.90	(0.74, 1.11)	0.3
Already has children	No	1		
	Yes	0.41	(0.08, 1.58)	0.3
MODEL 3				
Marital status	No	1		
	Yes	0.29	(0.07, 0.90)	0.03 *
Already has children	No	1		
	Yes	0.38	(0.09, 1.19)	0.1
Model 4				
Age	Per 5-year increase	0.99	(0.80, 1.22)	0.9
Marital status	No	1		
	Yes	0.30	(0.07, 0.99)	0.05 *
Already has children	No	1		
	Yes	0.40	(0.07, 1.61)	0.26
MODEL 5				
Age	Per 5-year increase	0.91	(0.72, 1.17)	0.5
Marital status	No	1		
	Yes	0.44	(0.10, 1.60)	0.3
Already has children	No	1		
	Yes	0.55	(0.11, 2.34)	0.6
Race and ethnicity	Non-Hispanic White	1		
	Non-Hispanic Asian	0.91	(0.02, 11.93)	1.0
	Non-Hispanic Black	3.06	(1.04, 9.02)	0.04 *
	Non-Hispanic Other	1.39	(0.12, 10.32)	1.0
	Hispanic Latino	1.51	(0.32, 5.95)	0.7
MODEL 6				
Age	Per 5-year increase	1.03	(0.75, 1.42)	0.9
Marital status	No	1		
	Yes	0.43	(0.09, 1.66)	0.3
Already has children	No	1		
	Yes	0.48	(0.07, 2.24)	0.5
Employment ^3^	Full-Time	1		
	Part-Time	2.81	(0.81, 9.80)	0.1
	Student	2.47	(0.78, 8.28)	0.1
	Disability or Unemployed	4.10	(1.37, 12.88)	0.01 *
MODEL 7				
Age	Per 5-year increase	0.97	(0.74, 1.28)	0.8
Marital status	No	1		
	Yes	0.31	(0.05, 1.21)	0.1
Already has children	No	1		
	Yes	0.61	(0.11, 2.57)	0.7
Education	≤High School or GED	1		
	Some College or Associates	2.51	(0.90, 7.80)	0.08
	Bachelors	1.10	(0.27, 4.45)	1.0
	Master’s or PhD	1.22	(0.20, 6.69)	1.0
MODEL 8				
Age	Per 5-year increase	0.87	(0.67, 1.13)	0.3
Marital status	No	1		
	Yes	0.47	(0.10, 1.70)	0.3
Already has children	No	1		
	Yes	0.54	(0.08, 2.47)	0.6
PROMIS50	<50	1		
	≥50	0.52	(0.24, 1.11)	0.1

^1^ Desire or unsure about fertility preservation vs. do not desire. ^2^ Exact logistic regression used when warranted due to small cell size. ^3^ Model excluded n = 6 retired individuals to facilitate convergence. * *p* ≤ 0.05.

## Data Availability

Some or all datasets generated during and/or analyzed during the current study are not publicly available but are available from the corresponding author on reasonable request.

## Supplementary Materials

https://www.mdpi.com/article/10.3390/jcm14176175/s1, Duke Adult Gender Medicine: Initial Visit Intake Questionnaire.
