# Supplementary material for: Interest in Fertility Preservation Among Adults Seen at a Gender Care Clinic
Source: J Clin Med. 2025 Sep 1;14(17):6175. doi: 10.3390/jcm14176175 (PMC12429561; doi:10.3390/jcm14176175)
Supplement: Supplementary file 1 [file jcm-14-06175-s001.zip › jcm-3783420-supplementary.pdf]

Duke Adult Gender Medicine: Initial Visit Intake Questionnaire

Visit Date:______________

Patient name (Used):	
Patient name (Legal, if different):	
Zip code of current residence:	
Referring provider:	

Pronouns (Check all that apply):

☐ He/Him/His ☐ She/Her/Hers ☐ They/Them/Theirs
☐ Name Only ☐ Not listed: _________________________

What gender(s) do you identify with? (Check all that apply)

☐ Female (cis gender) ☐ Genderqueer or Genderfluid ☐ Male (cis gender)
☐ Non-Binary ☐ Transgender Male ☐ Transgender Female
☐ Choose not to disclose ☐ Not listed:___________________________

Current Legal Gender (Registered with your health insurance—Check one):

☐ Female ☐ Male ☐ X ☐ Not listed: _____________________________

Sex Assigned at Birth (Check one):

☐ Female ☐ Intersex ☐ Male ☐ Unknown ☐ Not listed: ________________

Do you identify as (Check all that apply):

☐ Asexual ☐ Bisexual
☐ Gay ☐ Lesbian
☐ Pansexual ☐ Queer
☐ Straight/heterosexual ☐ I don't currently use a label for my sexuality
☐ Choose not to disclose ☐ Not listed:_______________________________

Goals for initial visit:

____
____
____
____

Comments:

____
____

Gender Transition History:

At approximately what age did you realize that your gender identity was different from the one you were assigned at birth?

______________ years old

Support System:

Which of the following groups are supportive of your gender identity? (Check all that apply)

☐ Parents ☐ Siblings ☐ Spouse ☐ Children ☐ Friends

☐ Extended family members ☐ Co-workers ☐ None of the above

Social Transition:

Have you transitioned socially in any of the following ways? (Check all that apply)

	Driver's License / State ID	Birth Certificate	Social Security Card	Passport	Student ID	Military ID	Not Listed: _________
a) Name Change on Identity Documents	☐	☐	☐	☐	☐	☐	☐
b) Gender Marker Change on Identity Documents	☐	☐	☐	☐	☐	☐	☐

	Family	Intimate Partner(s)	Friends	Co-workers
c) Asked others to use your new name	☐	☐	☐	☐
d) Asked others to use your new pronouns	☐	☐	☐	☐

e) ☐ Wardrobe change

f) ☐ Change in hairstyle

g) ☐ Finding social support groups

h) ☐ Not listed: ___________________________

i) ☐ No, I have not taken any steps toward social transition

Future Social Goals for Gender Transition: (check all that apply)							
	Driver's License / State ID	Birth Certificate	Social Security Card	Passport	Student ID	Military ID	Not Listed: __________
a) Name Change on Identity Documents	☐	☐	☐	☐	☐	☐	☐
b) Gender Marker Change on Identity Documents	☐	☐	☐	☐	☐	☐	☐
	Family	Intimate Partner(s)	Friends	Co-workers			
c) Ask others to use your new name	☐	☐	☐	☐			
d) Ask others to use your new pronouns	☐	☐	☐	☐			

e) ☐ Wardrobe change

f) ☐ Change in hairstyle

g) ☐ Find social support groups

h) ☐ Not listed: ____________________________

i) ☐ None of the above

Notes (need support coming out, need support navigating transition services, etc):

____

____

Physical Transition (non-medical):

Have you transitioned physically in any of the following ways (Non-medical, excluding surgical procedures or medications, check all that apply)

☐ Vocal coaching
☐ Chest binding
☐ Packing
☐ Tucking
☐ Padding
☐ Hair removal
☐ Not listed: ____________________________
☐ No, I have not taken any steps toward physical transition.

Future Goals for Physical Gender Transition: (Non-medical, excluding surgical procedures or medications, check all that apply)
☐ Vocal coaching<br>☐ Chest binding<br>☐ Packing<br>☐ Tucking<br>☐ Padding<br>☐ Hair removal<br>☐ Not listed: ___________________________<br>☐ None of the above

Medical Transition:

Medical Goals for Gender Transition: (Either now, or in the future—check all that apply)
☐ Hormone Replacement Therapy (HRT)<br>☐ Breast Reduction/augmentation (Top Surgery)<br>☐ Gender Affirmation Surgeries (Bottom Surgery, includes hysterectomy)<br>☐ Gender Affirming Cosmetic Surgeries (Such as Facial Feminization Surgery)<br>☐ None of the above

Are you currently on Hormone Replacement Therapy (HRT)?
☐ Yes ☐ No
Have you ever taken HRT while under the care of a licensed clinical provider (i.e. doctor)?
☐ Yes ☐ No
Have you ever taken HRT without a prescription and/or without the care of a licensed clinical provider?
☐ Yes ☐ No

Please list ALL HRT medications you currently or have previously used (if applicable):

**Medication Name**	**Current (or most recent) Dose**	**Start date** *MM/YY*	**End Date** (if applicable) *MM/YY*

Have you ever used silicone or other injectable fillers for body contouring without the supervision of a medical provider?
☐ Yes ☐ No
Have you undergone gender affirming surgeries (such as "Top Surgery", "Bottom Surgery", or "Facial Feminization Surgery")?
☐ Yes ☐ No

If yes, please describe below:

**Surgical Procedure Name**	**Date**<br>*MM/YY*	**Please list any complications.**<br>*Write "none" if no complications from surgery.*

Social History:

Education & Occupation:

What is the highest level of school you have completed or the highest degree you have received?
☐ 1st grade ☐ 2nd grade ☐ 3rd grade ☐ 4th grade ☐ 5th grade ☐ 6th grade ☐ 7th grade
☐ 8th grade ☐ 9th grade ☐ 10th grade ☐ 11th grade ☐ 12th grade ☐ High school graduate
☐ GED or equivalent ☐ Some college, no degree ☐ Associate degree: Academic program
☐ Associate degree: Occupational, technical, or vocational program ☐ Bachelor's degree (e.g., BA, AB, BS)
☐ Master's degree (e.g., MA, MS, MEng, MEd, MSW, MBA) ☐ Doctorate
☐ Professional school degree (e.g., MD, DDS, DVM, JD) ☐ Never attended school ☐ Prefer not to answer

Which of the following describe your current employment status? (Check all that apply)
☐ Employed full-time
☐ Employed part-time
☐ Self-employed
☐ Active duty military
☐ Student
☐ Disability
☐ Unemployed
☐ Retired

What is your current occupation? (Check all that apply)		
☐ Architecture & Engineering	☐ Business & Financial Operations	☐ Management
☐ Sales & Related	☐ Life, Physical, & Social Science	☐ Community & Social Service
☐ Installation, Maintenance, & Repair	☐ Armed forces occupations	☐ Healthcare Support
☐ Personal Care & Service	☐ Farming, Fishing, & Forestry	☐ Healthcare Practitioners & Technical
☐ Buliding & Grounds Cleaning/ Maintenance	☐ Art, Design, Entertainment, Sports, & Media	☐ Protective Service
☐ Construction & Extraction	☐ Food Preparation & Serving Related	☐ Office & Administrative Support
☐ Legal	☐ Production	☐ Computer & Mathematical
☐ Education, Training, & Library	☐ Transportation & Materials Moving	
☐ Not listed:____		☐ Prefer not to answer

Smoking:

Have you smoked cigarettes?	
☐ Current smoker (every day)	☐ Current smoker (some days)
☐ Former smoker If so, quit date:________	
☐ Never smoker (with passive smoke exposure)	☐ Never smoker
Current or former smokers:	
How many packs per day?	How many years of smoking?
_____ packs per day	_____ years
Do you vape?	
☐ Yes ☐ Not currently	☐ Never

Substance Use:

Do you use intravenous (IV) drugs?			
☐ Yes, within the last month	☐ Not currently (1-6 months ago)	☐ Not currently (> 6 months ago)	☐ Never
Do you use any other illicit or non-prescription substances?			
☐ Yes, within the last month	☐ Not currently (1-6 months ago)	☐ Not currently (> 6 months ago)	☐ Never

If yes (within the last month), please check the substances you have used in the last month (Check all that apply):<br>*Note: For prescription medications, please report non-medical use only*					
☐ Marijuana	☐ Inhalants	☐ Methamphetamine	☐ Hallucinogens	☐ LSD	☐ Crack cocaine
☐ Cocaine	☐ MDMA	☐ Heroin	☐ Tranquilizers	☐ Psychotherapeutics	
☐ Sedatives	☐ Stimulants	☐ Pain relievers	☐ CBD	☐ Not listed:______________________	

Alcohol:

Do you drink alcohol?
☐ Yes ☐ Not currently ☐ Never
How often do you have a drink containing alcohol?
_____ days per week

A standard drink is equal to:

- *12 ounces of regular beer, which is usually about 5% alcohol*
- *8-9 ounces of malt liquor, which is usually about 7% alcohol*
- *5 ounces of wine, which is typically about 12% alcohol*
- *1.5 ounces of distilled spirits or liquor, which is about 40% alcohol*

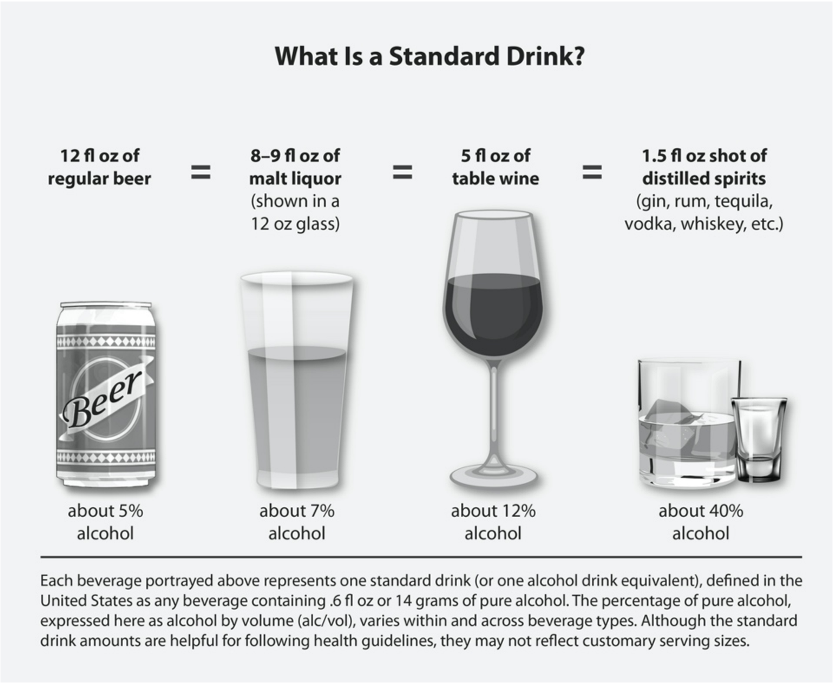

https://www.niaaa.nih.gov/alcohols-effects-health/overview-alcohol-consumption/what-standard-drink

How many standard drinks do you have on a typical <u>day</u> when you are drinking?
________ standard drinks
How many standard drinks do you have in a typical <u>week</u>?
standard drinks of wine: _______ # standard drinks of beer: ________ # standard drinks of spirits/liquor: ________

Relationships:

Please describe your current relationship status (Check all that apply):

☐ Single	☐ Married
☐ Divorced	☐ Legally Separated
☐ In monogamous relationship	☐ In polyamorous relationship(s)
☐ Live with my partner(s)	☐ Live separately from my partner(s)
☐ Not listed: ____	

Who do you **live with**?

Do not give names

Parents:	_____	Cousins:	_____
Step-parents:	_____	Grandparents:	_____
Siblings:	_____	Other Family:	_____
Step-siblings:	_____	Friends:	_____
Spouse/Partner:	_____	Roommates:	_____
Aunts/Uncles:	_____	Other Non-family:	_____

If you have children, what are their ages?
Do not give names -- list ages rounded to the nearest year

Child #1: ______	Child #4: ______	Child #7: ______	Child #10: ______
Child #2: ______	Child #5: ______	Child #8: ______	Child #11: ______
Child #3: ______	Child #6: ______	Child #9: ______	Child #12: ______

Social Issues:

Do any of the following apply to you? (Check all that apply)

☐ Problems with family or friends
☐ Emotional problems
☐ Occupational problems
☐ Housing problems
☐ Economic problems
☐ History of traumatic experiences (physical, sexual, emotional, psychological, or other)
☐ Problems with access to health care services
☐ Problems related to interaction with the legal system/crime
☐ Problems related to adjusting to new environment and/or country
☐ Other psychosocial and environment problems
☐ Substance use problems
☐ None of the above

Sexual and Reproductive History:

Have you ever had oral, vaginal, or anal sex?

☐ Yes, in the last 6 months ☐ Yes, not in the last 6 months ☐ No ☐ Prefer not to answer

If yes, which of your body parts have you ever used for sex?

☐ Penis (penetrative sex or receptive oral sex)
☐ Mouth (perform oral sex on someone else's genitals or anus)
☐ None
☐ Vagina (penetrative sex or receptive oral sex)
☐ Anus (penetrative sex or receptive oral sex)
☐ Prefer not to answer

With which body parts have you had unprotected sex (without a barrier method such as a condom or dental dam) in the last 6 months? (Check all that apply)

☐ Penis (penetrative sex or receptive oral sex)
☐ Mouth (perform oral sex on someone else's genitals or anus)
☐ None
☐ Vagina (penetrative sex or receptive oral sex)
☐ Anus (penetrative sex or receptive oral sex)
☐ Prefer not to answer

In the last 6 months, how many sexual partners have you had?

______ partners

Have you ever been told by a provider that you have any of the following?
Select one per row

	Yes, in the last 6 months	Yes, greater than 6 months ago	Never	Prefer not to answer
Gonorrhea	☐	☐	☐	☐
Chlamydia	☐	☐	☐	☐
Herpes (genital only)	☐	☐	☐	☐
Syphilis	☐	☐	☐	☐
HIV	☐	☐	☐	☐
Genital Warts	☐	☐	☐	☐
Trichomoniasis	☐	☐	☐	☐
HPV Positive or Abnormal Pap Smear	☐	☐	☐	☐

Please check all of the following statements that are true for you in the **last 6 months**:

☐ I have had sex with more than one person.
☐ I have had sex with someone who has a penis who has had sex with another person who has a penis.
☐ I have had a partner who uses IV drugs.
☐ I have had sex with someone who I know is infected with HIV.
☐ I have had sex with someone who I am unsure of their HIV status.
☐ I have had sex in exchange for something (drugs, money, rent, etc).
☐ I (or my partner) took emergency contraception to prevent pregnancy after sex.
☐ I took a medicine to prevent HIV after a risky event (Post Exposure Prophylaxis for HIV)
☐ None of the above is true for me over the past 6 months.
☐ I prefer not to answer this question.

Have you ever been tested for HIV?

☐ Yes ☐ Not sure ☐ No

If yes, what was the result of your most recent HIV test?

☐ HIV positive ☐ Not sure ☐ HIV negative

Do you wish to be screened for sexually transmitted infections and HIV at your visit?

☐ Yes ☐ Not sure ☐ No

Are you interested in PrEP? (Pre-exposure prophylaxis for HIV)

☐ Yes ☐ Not sure ☐ No ☐ Already on PrEP ☐ I would like to learn more about PrEP

Gynecologic History: (If applicable)

Age when period started: _______ years old

Last menstrual period: _______________ (MM/DD/YY) ☐ Postmenopausal

Average number of days between periods: _____ days

Do you have regular monthly periods?

☐ Yes ☐ Not sure ☐ No

Have there been any recent changes in your period?

☐ Yes ☐ Not sure ☐ No

Number of pads/tampons used on day of heaviest flow: _____

Menstrual Cramps:

☐ None ☐ Mild ☐ Moderate ☐ Severe

Do you have a history of male-pattern hair growth (face and body)?

☐ Yes ☐ Not sure ☐ No

Do you have a history of male-pattern scalp hair loss?

☐ Yes ☐ Not sure ☐ No

Do you have a history of excess acne?

☐ Yes ☐ Not sure ☐ No

Do you use any of the following for birth control or any other reason?

Check all that apply

☐ OCP (oral contraceptive pill)	☐ Condoms	☐ Diaphragm
☐ Vaginal ring (NuvaRing)	☐ IUD	☐ Spermicide
☐ Arm implant (Nexplanon/Implanon)	☐ Patch	☐ Cervical Cap
☐ Injection (Depo-Provera)	☐ Sponge	☐ Rhythm
☐ Surgical (vasectomy, hysterectomy, tubes tied)	☐ Coitus interruptus (pulling out)	☐ Post-menopause
☐ Abstinence (no sex)	☐ Not listed:_________________	☐ None

Pregnancy History: (if applicable)

Total number of pregnancies: _______	Number of miscarriages: ________	Number of biological children: ______
Number of full-term births: _______	Number of ectopic pregnancies: _____	Number of adopted children: ________
Number of preterm births: ________	Number of abortions: ______	Number of stepchildren: ________

Many treatments for transgender folks can impair your ability to produce sperm or become pregnant, though steps can be taken to preserve your fertility.

Are you interested in preserving your fertility?

☐ Yes ☐ Not sure ☐ No

If no or unsure, what are the possible reasons you are NOT interested in preserving your fertility? (Check all that apply)

☐ Too expensive ☐ Do not want biological children ☐ Prefer to adopt

☐ Hope to still have biological children even after hormones have been started

☐ The process of fertility preservation will cause too much distress and worsen gender dysphoria

☐ Not listed: ____

Personal Medical History:

Medical Condition	Yes	No	Date of Diagnosis (MM/YY) / Treatment / Important Details
Blood clot			
Breast cancer			
Ovarian Cancer			
Cervical Cancer			
Endometrial Cancer			
Prostate cancer			
Colon Cancer			
Kidney disease			
High potassium level			
Osteoporosis			
Hypertension (high blood pressure)			
Diabetes (high blood sugar)			
Heart attack			
Congestive heart failure			
High cholesterol			
High triglycerides			
Liver disease			
Migraine headaches			
Seizures			
Stroke			
Prolactinoma/Elevated Prolactin			
Gallstones			
High red blood cell count			
Sleep apnea			
Thyroid problems			
Polycystic Ovary Syndrome (PCOS)			
Substance abuse problem			
Eating Disorders Diagnosis			
Autism Spectrum Disorder			
Not listed:			
Not listed:			
Not listed:			

Comments:

____

____

Health Maintenance Screenings:

Cervical Cancer Screening (pap smear/HPV testing):

Cervical cancer screening can include a pap smear or HPV (human papillomavirus) testing, which are sometimes done together

Have you ever had a pap smear or HPV test?

☐ Yes ☐ No ☐ Not sure ☐ Does not apply

If yes:

Date of last pap smear or HPV test: Results:

______________________ ☐ Normal / Negative ☐ Abnormal / Positive

Have you ever had an abnormal pap smear or tested positive for HPV?

☐ Yes Date____________ ☐ No

Breast Cancer Screening (Mammogram):

Have you ever had a mammogram?

☐ Yes ☐ No ☐ Not sure ☐ Does not apply

If yes:

Date of last mammogram: Results:

______________________ ☐ Normal ☐ Abnormal

Have you ever had an abnormal mammogram?

☐ Yes Date____________ ☐ No

Osteoporosis Screening (Bone Density Scan or DEXA):

Have you ever had a bone density scan?

☐ Yes ☐ No ☐ Not sure

If yes:

Date of last bone density screen: Results:

______________________ ☐ Normal ☐ Osteopenia ☐ Osteoporosis

Have you ever been on treatment for osteoporosis?

☐ Yes ☐ No ☐ Not sure

Colon Cancer Screening:

Have you ever been screened for colon cancer with any of the following methods? (Check all that apply)

☐ Colonoscopy ☐ Fecal occult blood test ☐ FIT test ☐ Cologuard

☐ No ☐ Not sure

If multiple:

What was the last method of colon cancer screening you had? (Check one)

☐ Colonoscopy ☐ Fecal occult blood test ☐ FIT test ☐ Cologuard

Date of last colon cancer screening: Results:

_____________________ ☐ Normal ☐ Abnormal

Prostate Cancer Screening (PSA Test):

Have you ever had a PSA test?

☐ Yes ☐ No ☐ Not sure ☐ Does not apply

If yes:

Date of last PSA: Results:

_____________________ ☐ Normal ☐ Abnormal

Comments:

____

____

Other Past Surgeries:

Please list any other past surgeries you have had below:

Surgery	Date *(best estimate)*

Mental Health History:

Have you ever planned and/or attempted to kill yourself?

☐ Yes ☐ No If yes, how many attempts? ______ attempts

Have you ever deliberately harmed yourself without the intent to die?

☐ Yes ☐ No If yes, how many times? ______ times

Mental Health Providers:

Do you currently see a mental health provider (therapist, counselor, psychiatrist, psychologist, clinical social worker, etc.) for therapy or for medications?

☐ Yes ☐ No

In the table below, please list the provider you see for therapy, for medications or for both.

**Name of Provider/Practice, Email Address, and Phone Number**	**Do they provide mediations?** *Y/N*	**How long with this provider?** *(# of years)*	**Do they provide therapy?** *Y/N*

Mental Health Diagnoses:

Have you ever been given a psychiatric diagnosis by a licensed mental health provider?

☐ Yes ☐ No

In the table below, please list any psychiatric diagnoses you have been given by a licensed mental health provider and if you are currently receiving treatment for the diagnosis.

**Diagnosis**	**When Diagnosed?** *(best estimate)*	**Do you receive treatment?** *Y/N*	**Has there been improvement?** *Y/N*

Have you ever experienced any psychiatric hospitalizations?

☐ Yes ☐ No

In the table below, please list any psychiatric hospitalizations you've experienced

**Name of Hospital**	**Admission Date** *(best estimate)*	**What for?**	**Length of Stay** *(nights)*

Comments:

____

____

Family History:

Has any member of your family ever had the following? (Check all that apply)

**Family Member**	**Heart Disease**	**Stroke**	**Blood Clot**	**High Blood Pressure**	**High Cholesterol**	**Diabetes**	**Breast Cancer**	**Prostate Cancer**	**Ovarian Cancer**	**Not listed** *Specify below*
Mother										
Father										
Brother										
Sister										
Maternal Aunt										
Maternal Uncle										
Paternal Aunt										
Paternal Uncle										
Maternal Grandmother										
Maternal Grandfather										
Paternal Grandmother										
Paternal Grandfather										
Other family, not listed: *(do not give names)*										
Other family, not listed: *(do not give names)*										

Comments:

____

____

Review of Systems:

In the **past 6 months** have you experienced any of the following?

Check all that apply:

**Constitution**		**Cardiovascular**		**Musculoskeletal**	
Fever	☐	Chest pain	☐	Myalgias (muscle aches)	☐
Chills	☐	Palpitations	☐	Neck Pain	☐
Weight loss	☐	Orthopnea (difficulty breathing when laying flat)	☐	Back Pain	☐
Malaise / Fatigue	☐	Claudication (leg pain)	☐	Joint Pain	☐
Diaphoresis (unusual sweating)	☐	Leg Swelling	☐	Falls	☐
		PND (shortness of breath / coughing when sleeping)	☐		
**Skin**				**Endo/Heme/Allergy**	
Rash	☐	**Respiratory**		Easy Bruising or Bleeding	☐
Itching	☐	Cough	☐	Environmental Allergies	☐
		Hemoptysis (coughing up blood)	☐	Polydipsia (excess thirst)	☐
**HENT**		Sputum Production	☐		
Hearing Loss	☐	Shortness of Breath	☐	**Neurological**	
Tinnitus (ringing in ears)	☐	Wheezing	☐	Dizziness	☐
Ear Pain	☐			Headaches	☐
Ear Discharge	☐	**GI**		Tingling	☐
Nosebleeds	☐	Heartburn	☐	Tremor	☐
Congestion	☐	Nausea	☐	Sensory Change	☐
Sinus Pain	☐	Vomiting	☐	Speech Change	☐
Stridor (noisy breathing)	☐	Abdominal Pain	☐	Focal Weakness (one body part)	☐
Sore Throat	☐	Diarrhea	☐	Weakness	☐
		Constipation	☐	Seizures	☐
**Eyes**		Blood in Stool	☐	Loss of Consciousness	☐
Blurred Vision	☐	Melena (dark tarry stools)	☐		
Double Vision	☐			**Psychiatric**	
Photophobia (sensitivity to light)	☐	**GU**		Depression	☐
Eye pain	☐	Dysuria (pain with urination)	☐	Suicidal Ideas	☐
Eye Discharge	☐	Urgency (with urination)	☐	Substance Abuse	☐
Eye Redness	☐	Frequency (with urination)	☐	Hallucinations	☐
		Hematuria (blood in urine)	☐	Nervous / Anxious	☐
		Flank Pain (pain on sides between upper abdomen and back)	☐	Insomnia	☐
				Memory Loss	☐

Not listed: ____

Emotional Support – Calibrated Items

Please respond to each item by marking one box per row.

		Never	Rarely	Sometimes	Usually	Always
SSE-CaPS7	I have people who I can talk to about my health..	☐ 1	☐ 2	☐ 3	☐ 4	☐ 5
FSE31053x2	I have someone who will listen to me when I need to talk.................................	☐ 1	☐ 2	☐ 3	☐ 4	☐ 5
FSE31059x2	I have someone to confide in or talk to about myself or my problems	☐ 1	☐ 2	☐ 3	☐ 4	☐ 5
FSE31066x2	I have someone with whom to share my most private worries and fears	☐ 1	☐ 2	☐ 3	☐ 4	☐ 5
FSE31069x2	I have someone who understands my problems...	☐ 1	☐ 2	☐ 3	☐ 4	☐ 5
GS1x	I feel close to my friends..........................	☐ 1	☐ 2	☐ 3	☐ 4	☐ 5
GS2x	I get emotional support from my family...	☐ 1	☐ 2	☐ 3	☐ 4	☐ 5
SS11x	I have someone who makes me feel needed	☐ 1	☐ 2	☐ 3	☐ 4	☐ 5
SS12x	I have someone who makes me feel appreciated	☐ 1	☐ 2	☐ 3	☐ 4	☐ 5
SSE-CaPS6	I have someone I trust to talk with about my feelings..	☐ 1	☐ 2	☐ 3	☐ 4	☐ 5
SSQ1x	I have people who care about what happens to me	☐ 1	☐ 2	☐ 3	☐ 4	☐ 5
SSQ2x	I get love and affection	☐ 1	☐ 2	☐ 3	☐ 4	☐ 5

© 2008-2012 PROMIS Health Organization and PROMIS Cooperative Group

		Never	Rarely	Sometimes	Usually	Always
SSQ3x2	I have someone to talk with when I have a bad day	☐ 1	☐ 2	☐ 3	☐ 4	☐ 5
SSQ4x2	I have someone I trust to talk with about my problems	☐ 1	☐ 2	☐ 3	☐ 4	☐ 5
UCLA16x2	I feel there are people who really understand me	☐ 1	☐ 2	☐ 3	☐ 4	☐ 5
UCLA19x3	There are people I can talk to..................	☐ 1	☐ 2	☐ 3	☐ 4	☐ 5

© 2008-2012 PROMIS Health Organization and PROMIS Cooperative Group

Center for Sexual Health
1300 S 2nd St Suite 180
Minneapolis, MN 55454
Phone: 612.625.1500
Fax: 612.626.8311
http://www.umphysicians.org

Program in Human Sexuality
UNIVERSITY OF MINNESOTA

Body Image Scale-Gender Spectrum (BIS-GS)

DIRECTIONS: Choose two answers for each body part. (1) Check **the most appropriate box** as to how you feel for each specific body part, and (2) choose yes or no as to whether you would want to change each body part if it was possible through medical or surgical treatment.

If you do NOT have the body part, first choose DH (for "don't have), then check the most appropriate box as to how you feel about NOT having that body part. Then choose yes or no as to whether you would want to change that body part if it was possible through medical or surgical treatment.

		DH (Don't Have)	Very Satisfied (1)	Satisfied (2)	Neutral (3)	Dissatisfied (4)	Very Dissatisfied (5)	Change?
1	Nose	DH	1	2	3	4	5	YES | NO
2	Shoulders	DH	1	2	3	4	5	YES | NO
3	Hips	DH	1	2	3	4	5	YES | NO
4	Chin	DH	1	2	3	4	5	YES | NO
5	Calves	DH	1	2	3	4	5	YES | NO
6	Breasts	DH	1	2	3	4	5	YES | NO
7	Hands	DH	1	2	3	4	5	YES | NO
8	Adam's apple	DH	1	2	3	4	5	YES | NO
9	Vagina	DH	1	2	3	4	5	YES | NO
10	Scrotum	DH	1	2	3	4	5	YES | NO
11	Height	DH	1	2	3	4	5	YES | NO
12	Thighs	DH	1	2	3	4	5	YES | NO
13	Arms	DH	1	2	3	4	5	YES | NO
14	Eyebrows	DH	1	2	3	4	5	YES | NO
15	Clitoris	DH	1	2	3	4	5	YES | NO
16	Penis	DH	1	2	3	4	5	YES | NO
17	Waist	DH	1	2	3	4	5	YES | NO
18	Muscles	DH	1	2	3	4	5	YES | NO

McGuire, J., Spencer, K., Rider, G.N., & Berg, D. (2016). Body image scale-gender spectrum. [Adapted from: Lindgren, T.W. & Pauly, I.B. Body Image Scale. *Arch Sex Behav* (1975) 4: 639. doi:10.1007/BF01544272] *Unpublished, University of Minnesota*.

©Program in Human Sexuality, Regents of the University of Minnesota

		DH (Don't Have)	Very Satisfied (1)	Satisfied (2)	Neutral (3)	Dissatisfied (4)	Very Dissatisfied (5)	Change?
19	Buttocks	DH	1	2	3	4	5	**YES | NO**
20	Facial Hair	DH	1	2	3	4	5	**YES | NO**
21	Face	DH	1	2	3	4	5	**YES | NO**
22	Weight	DH	1	2	3	4	5	**YES | NO**
23	Biceps	DH	1	2	3	4	5	**YES | NO**
24	Ovaries-uterus	DH	1	2	3	4	5	**YES | NO**
25	Testicles	DH	1	2	3	4	5	**YES | NO**
26	Hair (on head)	DH	1	2	3	4	5	**YES | NO**
27	Voice	DH	1	2	3	4	5	**YES | NO**
28	Feet	DH	1	2	3	4	5	**YES | NO**
29	Figure	DH	1	2	3	4	5	**YES | NO**
30	Body Hair	DH	1	2	3	4	5	**YES | NO**
31	Chest	DH	1	2	3	4	5	**YES | NO**
32	Appearance	DH	1	2	3	4	5	**YES | NO**
33	Stature	DH	1	2	3	4	5	**YES | NO**

Suggested Citation: McGuire, J., Spencer, K., Rider, G.N., & Berg, D. (2016). *Body Image Scale-Gender Spectrum*. Unpublished, University of Minnesota.
Adapted from: Lindgren, T.W. & Pauly, I.B. *Body Image Scale*. Arch Sex Behav (1975) 4: 639. doi:10.1007/BF01544272)]

©Program in Human Sexuality, University of Minnesota

GAD-7 Anxiety

**Over the last two weeks, how often have you been bothered by the following problems?** *(Use "✔" to indicate your answer")*	**Not at all**	**Several days**	**More than half the days**	**Nearly every day**
1. Feeling nervous, anxious or on edge	0	1	2	3
2. Not being able to stop or control worrying	0	1	2	3
3. Worrying too much about different things	0	1	2	3
4. Trouble relaxing	0	1	2	3
5. Being so restless that it is hard to sit still	0	1	2	3
6. Becoming easily annoyed or irritable	0	1	2	3
7. Feeling afraid as if something awful might happen	0	1	2	3

If you checked off any problems, how difficult have these problems made it for you to do your work, take care of things at home, or get along with other people?

Not difficult at all	Somewhat difficult	Very difficult	Extremely difficult
☐	☐	☐	☐

From the Primary Care Evaluation of Mental Disorders Patient Health Questionnaire (PRIME-MD PHQ). The PHQ was developed by Drs. Robert L. Spitzer, Janet B.W. Williams, Kurt Kroenke and colleagues. For research information, contact Dr. Spitzer at rls8@columbia.edu. PRIME-MD® is a trademark of Pfizer Inc. Copyright© 1999 Pfizer Inc. All rights reserved. Reproduced with permission

PATIENT HEALTH QUESTIONNAIRE (PHQ-9)

**Over the last 2 weeks, how often have you been bothered by any of the following problems?** *(Use "✔" to indicate your answer")*	**Not at all**	**Several days**	**More than half the days**	**Nearly every day**
1. Little interest or pleasure in doing things	0	1	2	3
2. Feeling down, depressed, or hopeless	0	1	2	3
3. Trouble falling or staying asleep, or sleeping too much	0	1	2	3
4. Feeling tired or having little energy	0	1	2	3
5. Poor appetite or overeating	0	1	2	3
6. Feeling bad about yourself — or that you are a failure or have let yourself or your family down	0	1	2	3
7. Trouble concentrating on things, such as reading the newspaper or watching television	0	1	2	3
8. Moving or speaking so slowly that other people could have noticed? Or the opposite — being so fidgety or restless that you have been moving. around a lot more than usual	0	1	2	3
9. Thoughts that you would be better off dead or of hurting yourself in some way	0	1	2	3

10. If you checked off *any problems*, how *difficult* have these problems made it for you to do your work, take care of things at home, or get along with other people?	**Not difficult at all**	________
	**Somewhat difficult**	________
	**Very difficult**	________
	**Extremely difficult**	________

Copyright© 1999 Pfizer Inc. All rights reserved. Reproduced with permission. PRIME-MD® is a trademark of Pfizer Inc.
A2663 10-04-2005

Center for Sexual Health
1300 S 2nd St Suite 180
Minneapolis, MN 55454
Phone: 612.625.1500
Fax: 612.626.8311
http://www.umphysicians.org

Utrecht Gender Dysphoria Scale-Gender Spectrum

Instructions: For each question, select the response that best describes how much you agree with each statement.

Note: **Assigned sex** refers to the sex you were assigned at birth and **affirmed gender** refers to the gender that you feel most accurately represents your gender identity.

		**Disagree completely**	**Disagree**	**Neither agree nor disagree**	**Agree**	**Agree completely**
	**Question**	**1**	**2**	**3**	**4**	**5**
1	I prefer to behave like my affirmed gender	1	2	3	4	5
2	Every time someone treats me like my assigned sex I feel hurt	1	2	3	4	5
3	It feels good to live as my affirmed gender	1	2	3	4	5
4	I always want to be treated like my affirmed gender	1	2	3	4	5
5	A life in my affirmed gender is more attractive for me than a life in my assigned sex	1	2	3	4	5
6	I feel unhappy when I have to behave like my assigned sex	1	2	3	4	5
7	Living in my assigned sex feels positive for me	1	2	3	4	5
8	I enjoy seeing my naked body in the mirror	1	2	3	4	5
9	It is uncomfortable to be sexual in my assigned sex	1	2	3	4	5
10	Puberty felt like a betrayal	1	2	3	4	5

McGuire, J., Catalpa, J., Berg, D. & Spencer, K. (2016). The Utrecht gender dysphoria scale-gender spectrum. [Adapted from: Cohen-Kettenis, P.T. and van Goozen, S.H. The Utrecht Gender Dysphoria Scale. *J Am Acad Child Adolesc Psychiatry*. 1997; 36: 263–271] *Unpublished, University of Minnesota.*

©Program in Human Sexuality, Regents of the University of Minnesota

		Disagree completely	Disagree	Neither agree nor disagree	Agree	Agree completely
	**Question**	**1**	**2**	**3**	**4**	**5**
11	Physical sexual development was stressful	1	2	3	4	5
12	I wish I had been born as my affirmed gender	1	2	3	4	5
13	The bodily functions of my assigned sex are distressing for me (ie. erection, menstruation)	1	2	3	4	5
14	My life would be meaningless if I would have to live as my assigned sex	1	2	3	4	5
15	I feel hopeless if I have to stay in my assigned sex	1	2	3	4	5
16	I feel unhappy when someone misgenders me	1	2	3	4	5
17	I feel unhappy because I have the physical characteristics of my assigned sex	1	2	3	4	5
18	I hate my birth assigned sex	1	2	3	4	5
19	I feel uncomfortable behaving like my assigned sex	1	2	3	4	5
20.	It would be better not to live, than to live as my assigned sex	1	2	3	4	5

McGuire, J., Catalpa, J., Berg, D. & Spencer, K. (2016). The Utrecht gender dysphoria scale-gender spectrum. [Adapted from: Cohen-Kettenis, P.T. and van Goozen, S.H. The Utrecht Gender Dysphoria Scale . *J Am Acad Child Adolesc Psychiatry*. 1997; 36: 263–271] *Unpublished, University of Minnesota.*

©Program in Human Sexuality, Regents of the University of Minnesota
